# Predictors of pain intensity and persistence in a prospective Italian cohort of patients with herpes zoster: relevance of smoking, trauma and antiviral therapy

**DOI:** 10.1186/1741-7015-8-58

**Published:** 2010-10-11

**Authors:** Giustino Parruti, Monica Tontodonati, Cristina Rebuzzi, Ennio Polilli, Federica Sozio, Augusta Consorte, Adriana Agostinone, Francesco Di Masi, Gabriele Congedo, Domenico D'Antonio, Carla Granchelli, Claudio D'Amario, Carlo Carunchio, Lucio Pippa, Lamberto Manzoli, Antonio Volpi

**Affiliations:** 1 Infectious Disease Unit, Pescara General Hospital, Pescara, Italy; 2 Pain Management Clinic, Pescara General Hospital, Pescara, Italy; 3 Clinical Pathology Laboratory, Pescara General Hospital, Pescara, Italy; 4 Fondazione Onlus Camillo de Lellis per l'Innovazione e la Ricerca in Medicina, Pescara, Italy; 5 Clinical Microbiology Unit, Pescara General Hospital, Pescara, Italy; 6 Pescara Health District, Pescara, Italy; 7 Section of Epidemiology and Public Health, University of Chieti, Italy; 8 Department of Public Health, University of Rome "Tor Vergata", Rome, Italy

## Abstract

**Background:**

Herpes zoster (HZ) is a common disease, characterized by rash-associated localized pain. Its main complication, post-herpetic neuralgia (PHN), is difficult to treat and may last for months to years in the wake of rash resolution. Uncertainties remain as to the knowledge of predictors of HZ-related pain, including the role of antiviral therapy in preventing PHN in ordinary clinical practice. This prospective cohort study was aimed at investigating pain intensity at HZ presentation and its correlates, as well as the incidence of PHN and its predictors.

**Methods:**

Patients diagnosed with HZ were consecutively enrolled by a network of Italian General Practitioners and Hospital Units in the health district of Pescara, Italy, over two years. Uncertain cases were referred for microbiological investigation. Data were collected through electronic case report form (e-CRFs) at enrolment and at 1, 3, 6 and 12 months after enrolment. Pain intensity was coded on a five-degree semi-quantitative scale at each time point. PHN was defined as pain of any intensity during follow-up and quantified using an area-under-the-curve (AUC) method.

**Results:**

Four hundred and forty-one patients composed the final sample. Mean age was 58.1 years (SD = 20.4 years); 43.5% of patients were males; 7.9% did not receive prescription of antivirals. Intense/very intense pain at presentation was reported by 25.2% of patients and was significantly associated with female gender, older age, cigarette smoking, trauma and/or surgery at HZ site (logistic regression). PHN was diagnosed in 51.2% of patients at one month and in 30.0% of patients at three months. PHN was significantly associated with pain intensity at presentation, age, smoking, trauma and missed antiviral prescription (generalized estimating equations model). The same factors were also independent predictors of the overall pain burden as described by the AUC method (linear regression).

**Conclusions:**

Smoking, traumas and surgery at the HZ site emerged as new predictors of both HZ-related pain intensity and persistence, opening new perspectives in the prevention of HZ-related pain. An independent line of evidence was provided for the efficacy of antiviral therapy in preventing PHN and reducing total pain burden.

## Background

Herpes zoster (HZ) is a common disease, characterized by localized, monolateral pain and vesicular rash, due to the reactivation of latent varicella-zoster virus (VZV), with an incidence of 2.2 to 5.2 cases/1,000 patient-years [1-7]. Although HZ is often mild and self limiting [1,4,5,8,9], pain at presentation has been reported as intense or very intense in a remarkable proportion of patients [3,5]. Pain at presentation, however, has rarely been quantified, and its correlates have been poorly explored [10-12]. The main complication of HZ is represented by long lasting and/or relapsing pain at the site of rash, termed post-herpetic neuralgia (PHN), which is difficult to treat and may last for months to years, especially in people aged over 65 [9,13-16]. A better knowledge of the acute pain correlates, as well as of predictors of PHN may ultimately facilitate their prevention. Pain intensity at rash onset, age, rash severity, length of prodromal pain and cranial localization were more frequently reported as predictors of PHN [1,9,17-21]. At present, little is known on the possible role of trauma and surgery at the site of VZV reactivation [22-26]. Similarly, uncertainties remain on the role of antiviral therapy for both controlling acute pain and preventing PHN in ordinary clinical settings, because most data on antivirals in HZ came from trials selectively enrolling patients diagnosed ≤ 72 hours after disease onset [27]. As a consequence, the usefulness of antiviral therapy in HZ has been questioned until recently [4,5,8,27,28]. Finally, although smoking can have immunomodulatory effects and, therefore, influence the pathogenesis of latent reactivation infections [29], its association with PHN has never been formally investigated.

We carried out a prospective, monocentric cohort study, enrolling a large sample of consecutive unselected patients with HZ, in order to evaluate intensity of pain at presentation, pain persistence during follow-up and the overall pain burden, as well as several potential correlates of pain intensity at presentation and predictors of PHN.

## Methods

### Study population and design

A network was established among 41 general practitioners (GPs) in the district of Pescara, Italy. Between 1 May 2006, and 30 April 2008, GPs enrolled all incident cases of HZ observed. In addition to GPs, the Infectious Diseases (ID) Unit, the Dermatology Unit, and the Department of Pain Medicine and Palliative Care, shortly Pain Management Clinic (PMC) of the Pescara General Hospital, Pescara, Italy, reported consecutive data on HZ patients observed. Enrolling physicians were connected through a dedicated software, avoiding repeated enrolments. The study was approved by the local Ethics Committee. Enrolling physicians were requested to report prescribed antivirals and untreated cases; they were suggested to prescribe antivirals whenever active lesions were present, in accordance with current local trends in prescription. An informed consent was signed by patients at enrolment, and an e-CRF was filled in with demographic and clinical information. One or more pictures of lesions were also included for patients consenting to be pictured, to allow expert review. A one-year follow-up was planned for enrolled cases.

### Diagnosis of herpes zoster

Diagnosis of HZ was clinical whenever enrolling physicians felt it appropriate, with no additional evidence needed. Patients were enrolled whatever the stage of the rash and the time interval from the rash onset. The extent of the rash was codified as mild-moderate or severe according to the number of lesions (respectively, < 50 and ≥ 50), defined as papules, vesicles or crusted vesicles [19]. When GPs felt that laboratory investigation should be performed, uncertain cases were referred to the ID Unit for evaluation within 24 to 48 hours. Review of clinical data was provided, together with microbiological investigation by assays of both IgG and IgM VZV-specific antibodies, as well as by assays of plasma samples, vesicle eluates and other specimens as appropriate by Real Time VZV DNA PCR (Polymerase Chain Reaction, Nanogen^©, ^Italy) [30,31].

### Pain intensity at presentation

HZ patients were asked about pain severity at presentation, as well as at every further visit or phone call. Follow up visits or phone calls were performed at 1, 3, 6 and 12 months after enrolment. Pain intensity was coded on a five-degree semi-quantitative scale (including no pain, mild, moderate, intense or very intense pain). All patients reporting intense or very intense pain were granted immediate access to PMC. Use of analgesics (NSAIDs, paracetamol or tramadol) at enrolment was reported. Adverse events other than pain were classified according to the Common Terminology Criteria for Adverse Events, Version 3.0, USA [32].

### Qualitative and quantitative diagnosis of Post Herpetic Neuralgia

PHN was defined as the presence of pain of any intensity at follow-up visits, measured with the above described scale. At each follow-up visit or phone call patients were asked to report on the worst pain intensity experienced since their last visit or phone call, possibly specifying the time of occurrence within the interval. To take into account the total burden of pain in the individual patient, pain during follow-up was quantified using an area-under-the-curve (AUC) method, similar to that proposed by Coplan *et al. *[33]. Briefly, we combined measures of pain intensity and duration, and each patient's AUC was calculated as the sum of all areas obtained by multiplying the average of two consecutive pain scores by the number of days between the scores.

### Correlates of pain intensity at presentation and predictors of PHN

A number of potential correlates of pain intensity at presentation and predictors of PHN were evaluated: age; gender; familial status (single, married or divorced); educational level (lower, no education or primary school; higher, any diploma or degree); hypertension; diabetes mellitus; HCV and/or HIV infection; alcohol abuse (binge drinking or ≥ 80 grams daily); current or former cigarette smoking (current, ≥ 1 cigarette daily, for ≥ 5 years; former, if patients smoked as defined above for at least 5 years quitting ≤ 2 years before presentation); familial history of major cardiovascular events (occurred in first degree relatives aged ≤ 60 years); malignancies, neurological diseases, major depression or psychiatric illnesses, allergy (any past reaction to environmental, alimentary or cutaneous antigens). Clinical history was considered positive for trauma when patients recalled any type of trauma at the site of lesions (contusions, burnings, wounds, politraumas involving the reactivation site) up to six months ahead of enrolment. Surgical interventions at the site of lesions were considered whenever performed. Other potential predictors were: site of lesions, pain intensity at presentation, rash severity and prescribed NSAIDs. Finally, patients prescribed antivirals were compared to those not prescribed antiviral therapy for any reason.

### Data analysis

Initial descriptive statistics were used to analyse the distribution of PHN cases in the sample and according to all variables investigated. Logistic regression was used to evaluate potential independent predictors of intense/very intense pain at presentation (as compared to mild/moderate pain), as well as to investigate potential predictors of PHN at one or three months (two separate models). In all models, covariates were included in a stepwise forward process with the following inclusion criteria: *P *< 0.15 at univariate analysis; change in significant odds ratios (OR) > 20%; age and gender forced to entry. Standard post-estimation tests were used to assess final model validity (Hosmer-Lemeshow test for evaluation of the goodness of fit, area under the ROC curve for predictive power [34]), performing multicollinearity (using Spearman coefficients) and influential observation analyses (using standardized residuals, change in Pearson and deviance chi-square [35]), and testing for potential interaction and higher power terms. The model predicting pain at presentation was repeated excluding seven influential observations with no relevant differences. The models predicting PHN at one and three months were repeated excluding 14 and 11 influential observations, respectively, with no substantial variations. Separate logistic models for the time points 6 and 12 months were not fitted because of the small number of successes (n = 43 and n = 33, respectively), with high potential for overfitting. However, the data from 6- and 12-month visits were included in a generalized estimating equations (GEE) model, set as a repeated measures logistic regression (logit link and binomial family), which was performed to investigate the potential interactions between time and each of the potential PHN predictors [36]. The model was fitted assuming an exchangeable correlation structure, with robust standard errors (based upon sandwich estimator) [37]. Time was included in the model either as an ordinal variable or using dummy variables for each visit (a one-month visit being the reference category). Given that all dummy ORs were significantly associated with PHN and were quite linearly decreasing, time was treated as ordinal in the final model: the OR of time is thus to be interpreted as the variation in the odds of PHN for every visit increase (that is, the OR of PHN at the third, six-month visit vs the second, three-month visit). It is important to specify that the GEE model represented the main and most reliable analytical approach to investigate the potential predictors of PHN during follow-up.

To evaluate independent associations between pain burden and other variables under investigation, a linear regression analysis was used. The dependent variable of the model was AUC, which was transformed into its square root because of its skewed distribution (Shapiro-Wilk test). Because of the incomplete follow-up for some participants, the total follow-up duration was included as a covariate into the final model, whose validity was assessed as follows: the assumption of constant error variance was checked graphically, plotting Pearson residuals vs fitted values, and formally, using Cook and Weisberg's test for heteroskedasticity. High leverage observations were identified using Pearson, standardized and studentized residuals, Cook's D influence, Welsch distance and hat diagonal matrix [35]. High leverage observations were, however, < 5% of the total sample with all methodologies, with no appreciable changes on the final results after their exclusion (the final model was therefore based upon the total sample). All multivariate models reliability was also checked using bootstrap estimation with 500 replications. The bias-corrected bootstrap showed quite similar results for all models, thus bootstrapped estimates were not shown to avoid redundancy. Finally, in all multivariate analyses, missing values were < 5% of the total sample; multiple imputation procedures were thus avoided. Statistical significance was defined as a two-sided *P*-value < 0.05 for all analyses, which were carried out using Stata version 10.1 (Stata Corp., College Station, TX, USA, 2007).

## Results

Overall, 519 patients were enrolled, 68.0% by the GPs and 32.0% by the Hospital Units. Microbiological investigation was requested for 63 subjects (12.1%), and HZ was diagnosed in 28 of them. HZ was not confirmed for the remaining 35 patients, who were excluded. Forty-three subjects were also excluded because of diagnosis of other dermatological diseases by expert review of clinical and pictorial data (n = 6), insufficient data provided at the first visit (n = 9), consent withdrawal during follow-up (n = 28). As a consequence, 441 patients were followed for a mean of 335 days (SD = 82 days). Twenty-four (5.4%) patients were hospitalized for a mean of 9.3 (3.2) days; no patient died during follow-up. The baseline characteristics of the sample are summarized in Table 1. The mean age was 58.1 (20.4) years; median age was 63 years; 43.5% of patients were males. Rash was severe (> 50 vesicles) in 30.2% of the 278 evaluable patients. Median time between rash onset and enrolment was three days (Interquartile Range, IQR, 3), 56% of patients being enrolled by the third day from rash onset and 89.2% by the seventh day. For 63 (14.3%) of 433 patients in the final sample a possibly relevant comorbidity was reported: 39 solid tumours (8.8%), 10 leukemias or lymphomas (2.3%), 11 neurological disorders (including Alzheimer disease, multiple sclerosis, or peripheral neuropathies), and 6 mood disorders (all major depression). In accordance with our study protocol, 406 patients were prescribed antivirals on the same day of enrolment (acyclovir, n = 148; valacyclovir, n = 148; famcyclovir, n = 47; brivudin, n = 63); 35 patients (7.9%) received no prescription of antiviral drugs, 5 as a choice of the enrolling physician, 19 because of the fear of allergic reactions in poliallergic patients, 11 for very late presentation, that is very advanced rash evolution. An overlapping distribution, far from reaching any statistically significant difference, was observed for all variables in the final sample and in the 37 patients excluded due to insufficient data at inclusion or consent withdrawal during follow-up.

**Table 1:** Selected demographic, behavioural and clinical characteristics of the final sample of HZ patients.

Variables	Overall sample (n = 441)	PHN one-month definition (n = 226)	No PHN one-month definition (n = 210)	P*	PHN three-month definition (n = 130)	No PHN three-month definition (n = 304)	P*
*Demographic characteristics*							
Mean age in years (SD)	58.1 (20.4)	61.1 (18.8)	54.9 (21.7)	0.002	60.4(19.3)	57.0 (21.0)	0.12
	%	%	%		%	%	
Female gender	56.5	57.5	55.4	0.6	60.0	54.7	0.3
Married	76.4	79.6	73.2	0.10	83.1	73.3	0.02
Higher educational level	39.7	38.9	40.5	0.7	39.2	40.1	0.9
							
*Anamnesis*							
Hypertension	36.0	39.4	32.6	0.14	33.8	36.8	0.7
Diabetes mellitus (missing = 3)	9.1	10.7	7.5	0.2	8.6	9.1	0.9
HCV infection	1.4	2.2	0.5	0.11	1.5	1.3	0.8
HIV infection	1.1	1.8	0.5	0.2	1.5	1.0	0.6
Neoplasm (m = 8)	11.3	12.7	9.9	0.3	10.0	11.4	0.9
Neurological disorders	2.5	3.5	1.4	0.15	3.1	2.3	0.6
Psychiatric illnesses (m = 17)	1.4	1.4	1.5	0.9	0.0	2.1	0.10
Allergy	10.9	15.5	6.1	0.001	16.1	8.8	0.025
Trauma (m = 3)	16.0	24.5	7.0	< 0.001	31.8	9.5	< 0.001
Surgical intervention (m = 3)	31.0	41.5	20.1	< 0.001	46.9	24.5	< 0.001
Alcohol abuse (m = 7)	6.9	4.5	9.5	0.04	4.0	7.9	0.14
Current or former smoking (m = 2)	26.0	32.4	19.2	0.002	38.0	20.9	< 0.001
Familial CHD risk (m = 2)	25.1	29.9	20.0	0.017	31.2	22.2	0.045
							
*Clinical characteristics*							
*HZ dermatomeric district*							
Thoracic	49.8	49.1	50.5	0.8	46.9	51.3	0.4
Lumbar	22.9	23.4	22.4	0.8	25.4	21.2	0.3
Cranial	18.2	20.8	15.4	0.14	19.2	18.0	0.8
Cervical	9.1	6.6	11.7	0.07	8.5	9.5	0.7
							
*Pain intensity*							
Mild	40.5	30.1	51.6	< 0.001	26.1	46.9	< 0.001
Moderate	34.2	33.6	34.7	0.8	31.5	35.4	0.4
Intense	18.9	26.1	11.3	< 0.001	30.0	13.8	< 0.001
Very intense	6.4	10.2	2.3	< 0.001	12.3	3.9	0.001
							
> 50 vesicles (m = 163)	30.2	37.1	22.0	0.007	38.4	26.8	0.054
Missed prescription of antivirals	7.9	11.9	3.7	0.001	16.1	4.6	< 0.001
Use of analgesics in the acute phase	43.0	47.3	38.5	0.061	42.3	43.6	0.8

When no missing observations (m) are indicated, there were no missing values. * Chi-square test for categorical variables; t-test for continuous variables. Only the variables reported in the table have been included in the model.

### Correlates of pain intensity at presentation

Intense or very intense pain at presentation was reported by 25.2% of the patients (n = 111; Table 2). The logistic regression analysis showed that patients reporting intense or very intense pain at presentation were significantly more likely to be female, older, present or past cigarette smokers, with history of trauma and/or surgical intervention at the site of HZ. They also reported more frequently taking analgesics in the acute phase (all *P *< 0.05, Table 2). Rash severity could not be considered in multivariate models because 163 patients did not consent to pictorial documentation.

**Table 2:** Results of the logistic regression model predicting intense/very intense pain at presentation.

	Intense/Very intense pain (n = 111)	Mild/Moderate pain (n = 330)	OR (95% CI)	*P*
Mean age in years (SD)	65.4 (14.0)	55.6 (21.7)	1.02 (1.01 to 1.04)*	< 0.001
	%	%		
Female gender	69.4	52.1	2.50 (1.41 to 4.35)	0.002
Current/former smoking	33.3	23.5	2.00 (1.11 to 3.61)	0.021
Trauma	30.6	11.0	2.51 (1.30 to 4.84)	0.006
Surgical intervention	50.0	24.7	2.09 (1.21 to 3.62)	0.008
Use of analgesics in the acute phase	65.4	35.6	3.60 (2.17 to 5.98)	< 0.001

* One-year increase.

### Predictors of PHN and overall pain burden

**Figure 1:**
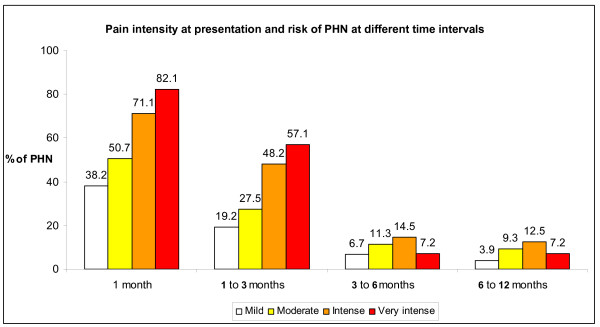
**Pain intensity at presentation and the risk of PHN at different time intervals**. Proportion of patients developing PHN at different time intervals according to pain intensity at presentation. Patients with PHN were 51.2% (n = 226) at one month after enrolment; 30.0% (n = 130) after one to three months; 9.8% (n = 43) after three to six months; 7.5% (n = 33) after 6 to 12 months.

Pain was reported by 51.2% of patients at one month after enrolment (n = 226); by 30.0% (n = 130) during the time interval one to three months; by 9.8% (n = 43) during the time interval three to six months; by 7.5% (n = 33) during the time interval 6 to 12 months. The frequency of PHN was clearly and tightly related to the semi-quantitative grading of pain at presentation at the one- and one- to three-month time points (Table 1, Figure 1). PHN at one month was as low as 37.8% in patients with mild pain at presentation, raising up to 82.1% in patients with very intense-intolerable pain. Similar results were observed for the three to six months and 6 to 12 months time intervals (Figure 1).

The results of the repeated measures analysis (GEE) investigating potential independent predictors of PHN during the follow-up are shown in Table 3. The associations observed at univariate analyses for age, pain at presentation, smoking, trauma and missed antiviral prescription (although borderline) were confirmed when controlling for potential confounders. Expectedly, time was highly and negatively associated with PHN, but none of the interaction terms between time and any PHN predictor achieved significance (neither the most clinically relevant one, missing antiviral therapy-time, which showed a *P-*value of 0.44).

**Table 3:** Results of the generalized estimating equations (GEE) model predicting PHN during the entire follow-up.

Variables	OR	(95% CI)	*P*
Age, ten-year increase	1.01	(1.00/1.02)	0.017
Female gender	0.91	(0.63/1.32)	0.6
Current/former smoking	1.50	(1.02/2.21)	0.039
Intense/very intense pain at presentation	1.85	(1.29/2.65)	0.001
Trauma	2.27	(1.48/3.46)	< 0.001
Surgical intervention	1.40	(0.96/2.02)	0.081
Missed antiviral prescription	1.46	(1.00/2.25)	0.049
Time, one-visit increase	0.73	(0.68/0.78)	< 0.001

OR = Odds Ratio

We also reported the results of separate logistic regression models investigating potential independent predictors of PHN at one month and three months (Table 4). The results of both models are mostly concordant with those of the repeated measures model, which is the main and most reliable analysis. These additional logistic models were made only to facilitate the comparison between our findings and those from previous studies, the majority of which used such time intervals.

**Table 4:** Results of the logistic regression models predicting PHN at one and one to three months.

	PHN at one month			PHN at one to three months		
Variables	OR	(95% CI)	*P*	OR	(95% CI)	*P*
Age, ten-year increase	1.01	(1.00 to 1.02)	0.032	1.01	(0.99 to 1.02)	0.4
Female gender	1.05	(0.68 to 1.63)	0.8	1.39	(0.84 to 2.30)	0.2
Current/former smoking	1.62	(0.98 to 2.67)	0.061	2.08	(1.22 to 3.55)	0.007
Intense/very intense pain at presentation	2.41	(1.43 to 4.04)	0.001	2.19	(1.32 to 3.65)	0.003
Trauma	2.22	(1.12 to 4.39)	0.022	2.53	(1.37 to 4.65)	0.003
Surgical intervention	1.60	(0.98 to 2.63)	0.062	1.33	(0.79 to 2.25)	0.3
Missed antiviral prescription	2.01	(1.01 to 4.46)	0.047	2.28	(1.04 to 4.98)	0.038

OR = odds ratio

An AUC was obtained for each patient. Age, pain intensity at presentation, smoking, missed antiviral prescription and trauma were significantly and independently associated with total pain burden as measured by the AUC in the final linear regression model (all *P *< 0.05, Table 5).

**Table 5:** Regression model predicting pain burden during follow-up.

Variables	Coefficient	(95% CI)	*P*
Age, 10-year increase	0.03	(0.01/0.05)	0.004
Female gender	0.68	(-0.21/1.59)	0.14
Current/former smoking	1.50	(0.47/2.53)	0.004
Intense/very intense pain at presentation	3.40	(2.37/4.43)	< 0.001
Trauma	1.58	(0.31/2.85)	0.015
Missed antiviral prescription	2.22	(0.63/3.82)	0.006

Pain burden was calculated by multiplying pain intensity for its duration, using the area-under-the-curve (AUC) method described in the text. The dependent variable AUC was transformed into its square root to approach normality.

## Discussion

Our prospective observational cohort study was aimed at investigating pain intensity at the onset of HZ and its correlates, as well as predictors of PHN and overall pain burden in real-life clinical settings. Our study was designed and executed independently of any restraint or support by pharmaceutical companies.

### Pain intensity at presentation and its correlates

A quarter of the enrolled sample presented with intense or very intense pain, according to the adopted semi-quantitative five-degree scale. This percentage was lower than the 41% rate of patients with severe or very severe pain observed by Chidiac *et al. *in their prospective cohort [3]. Other retrospective investigations were not aimed at quantifying zoster-associated pain (ZAP) at presentation and do not therefore provide a direct reference for this finding [1,2,4-7,17,18,20]. The proportion of patients with intense or very intense pain at presentation may have been overestimated in our study if very mild cases never requested medical assistance; there are no reasons, however, to believe that differences between possibly missed and included cases would be so relevant as to substantially modify our evaluation of the correlates of pain intensity at presentation as well as of PHN. Intense pain at presentation was independently related with age, female gender, present or former cigarette smoking, trauma and/or surgery at the site of HZ. Intense pain was also somewhat obviously associated with more frequent use of analgesics at enrolment.

Some data suggest that the perception of pain from myocardial ischemia is significantly less severe and delayed in the elderly compared to younger patients [38]. Furthermore, many clinical studies suggest a relative decrease in the frequency and intensity of pain symptoms associated with myocardial complaints, visceral infections, musculoskeletal conditions, and postoperative and malignant pain problems in adults of advanced age [39]. More closely related to our field, data from prospective studies have demonstrated that HZ patients with severe acute pain at presentation have an increased risk of developing PHN, but few studies have examined the relationships between acute pain severity and age. Some studies even showed that older age was not consistently associated with more intense acute pain [12]. Our results, however, were clear-cut significant: mean age was 55.6 years (SD = 21.7) in patients with low or moderate pain at presentation and 65.4 years (14.0) in those with intense of very intense pain; 54.9 years (21.7) in patients without PHN and 61.1 years (18.8) in patients with PHN, both with confidence intervals not overlapping at all. So they confirm those of Dworkin *et al*., 2001, indicating that older age and rash severity were associated with more severe acute pain assessed within 72 h of rash onset [12]. Trauma or surgery at the site of HZ has been already suggested as a predictor of VZV reactivation [22-26]. Our estimate of the proportion of patients reporting surgery in advance of HZ (31%) is somewhat higher than that reported by other authors, and this fact may be difficult to explain; it cannot be related to any selection bias, however, as patients were recruited by GPs or in medical wards of the local hospital. Therefore, it is likely that this potential correlate, which was searched in the medical history section at enrolment, may have been more systematically investigated in our setting than in others. In addition, surgical interventions have never been investigated as a possible correlate of pain intensity and persistence in HZ patients in a large cohort as ours [22-26]. Our finding of a remarkable association between surgery and trauma and pain intensity at presentation of HZ suggest that nerve fibre impairment secondary to these events may predispose to more intense pain. Female gender has already been associated with a more intense perception of pain in several other clinical conditions, and gender differences in clinical and experimental pain sensitivity and response to pain treatment have been thoroughly investigated [40]. The status of being a present or past smoker almost doubled the risk of intense pain in our sample. A potential role for smoking in chronic pain syndromes has been well documented [41]. Furthermore, smoking has been associated with frequent subclinical peripheral neuropathy in patients with chronic obstructive pulmonary disease [42,43]. These associations may be due to structural or functional impairment of pain transmission by chronic smoking habits [41]. Moreover, the association of smoking with pain intensity might be due to smoke-induced impairment of cell-mediated immunity, leading to more aggressive viral activity during VZV reactivation [29]. In our multivariate analyses, after several binomial and multinomial logistic models suggesting a similar association between current or past smoking and pain at presentation (data not shown), current and former smokers were joined as a single group opposed to non smokers. Our data would therefore suggest that the influence of chronic cigarette smoking on pain at presentation may persist even after quitting smoking. In any case, however, all the results on the association between smoking and pain require confirmation from further research, as we were not able to include in the analysis some potential confounders such as anxiety and depression, which have been associated to both higher HZ pain intensity and smoking [10,44,45].

### Predictors of PHN and total pain burden

The other major aim of our study was to consider and quantify pain persisting any longer than rash healing, looking for predictors of such persistence. Remarkable differences exist as to the time interval considered for the diagnosis of PHN [13-16,46]. Most authors so far set at three or six months the threshold for PHN to be diagnosed after rash healing or onset; in particular Oxman *et al. *defined PHN as ZAP rated as ≥ 3 on a 0-to-10 VAS scale and persisting or appearing > 90 days after the rash onset [46]. To account for ZAP restricted within this time frame, which involves over 30% of HZ patients, PHN was diagnosed elsewhere when pain persisted one month after rash onset or healing [8,14,47]. In the present study, pain was considered as a continuum and its total amount in the individual patient was quantified using our AUC method rather than choosing an arbitrary time threshold. To facilitate comparisons, however, we diagnosed PHN qualitatively using both the one-month and the one- to three-month thresholds. Indeed, also the qualitative three- to six-month threshold was considered and dropped, because of the yield of identical results (data not shown). We, however, used a GEE model as the main multivariate model to better account for pain relapsing at later time points. The accuracy of the AUC method that we used may not be as high as that used by Coplan and Oxman et al., who collected data concerning pain intensity much more frequently than we did [33,46]. We diagnosed PHN when pain of any degree persisted or relapsed at follow up visits. We allowed progressively longer time intervals among visits, up to six months between the last two visits. This may have reduced accuracy in detecting PHN, representing a limitation of our study design [48,49]. Pain in PHN, however, is long lasting and easy to interpret even by ordinary culture people [49,50]. This may have partly counterbalanced the underestimation of pain caused by longer time intervals between follow-ups in our series [50]. On the other hand, we may have overestimated PHN by the AUC method, as an intense pain experience in the beginning of a broad time-window may have been carried forward to the following distant endpoint. As the AUC method, however, confirmed results obtained with the logistic regression models and with the repeated measures multivariate analysis, a possible overestimation of pain persisting in a few patients is not likely to have biased our general conclusions.

A number of parameters have been proposed as predictors of PHN, pain intensity and age at presentation being the only ones consistently emerging in most of previous investigations [1,9,17-21]. We evaluated a large array of items in patients' history, clinical presentation and therapy, and observed a strong association of PHN with intense pain at presentation both at 1 month and one- to three-month intervals, confirming acute pain intensity as one of the most relevant predictors of pain duration [1,17,18]. Age was similarly confirmed, adding evidence that older age is associated not only with a higher risk of VZV reactivation, but also with a higher probability of pain persistence [1,9,12,17,18]. The aging PNS and CNS may indeed poorly tolerate damage to a reduced number of functional neurons, thus increasing the likelihood of PHN development and duration. Elderly persons are also likely to have concomitant diseases and medications for their management [51]. As to prodromal pain duration and rash severity, frequently reported as tight predictors of PHN in previous studies, these variables could not be included in our final models because of the high proportion of missing values; this likely represents one major limitation of our investigation. Three additional novel factors, however, were found tightly associated with the total amount of pain and PHN. First, a protective role of antiviral therapy, prescribed any time during rash evolution and whatever the drug of choice, emerged as statistically significant. So far, the role of antiviral therapy was mainly investigated in randomized controlled trials (RCT), including only patients diagnosed within 72 hours from onset of HZ [27,47], and its efficacy in preventing PHN has been recently questioned by a meta-analysis including most of the above mentioned trials [27]. Until recently, indeed, HZ has been frequently considered a self limiting disease [1,4,5,8,9] and antiviral therapy of debatable usefulness [4,5,18,27,28]. This may well be the case if the main aim of therapy would be reducing complications other than pain [52]. In the present cohort, in which the role of antiviral therapy was examined in current clinical practice, PHN was more frequent both at one and one- to three-month intervals both in univariate and multivariate analyses in the small proportion of patients not prescribed antivirals, as well as in the repeated measures multivariate analysis. Although the design of the present study does not allow to draw any firm conclusion about the effectiveness of antivirals with respect to pain, as an RCT would be the appropriate study design, our investigation confers an additional clue that antivirals may reduce the total burden of pain in the individual patient, even when prescribed beyond the 72-hour time threshold, as in half of patients in our series, in line with current clinical practice [10,21,47]. Second, major traumas were significantly associated with the total amount of pain and PHN in all statistical models. Traumas and work-related injuries have been already associated with an increased risk of HZ [22-24]. It may be plausible, therefore, that major traumas may induce local changes facilitating VZV reactivation in spinal ganglia and contributing also to more intense and durable pain. Clearly, such an hypothesis should be further investigated. Third, cigarette smoking emerged as a predictor of pain persistence at all investigated time points. This finding reinforces the significance of its above discussed association with pain intensity at presentation. The same hypothesized impairment of pain transmission pathways might be responsible for its persistence. Further investigations, however, including pathological and/or *in vitro *studies, will be necessary to demonstrate a causal linkage. Our survey included a very limited number of patients with comorbilities, which are known to increase the risk of HZ. Such few cases did not allow us to stratify our analyses for comorbid patients. However, our sample allows us to confirm previous data, suggesting that most of incident cases of HZ occur in otherwise healthy and relatively young adults [6,7].

## Conclusions

Our study provides new insights into the knowledge of pain intensity at presentation and pain persistence due to HZ in a large, monocentric, prospective cohort of unselected Caucasian patients. Cigarette smoking, traumas and surgery at the site of the HZ emerged as new predictors of both intensity and persistence of pain, all of which may well deserve further investigation. A new, independent line of evidence is provided as to the efficacy of antiviral therapy in reducing the incidence of PHN and the total burden of pain caused by HZ.

## Abbreviations section

AUC: area-under-the-curve; CNS: central nervous system; e-CRF: electronic case report form; GEE: generalized estimating equations; GP: general practioners; HZ: herpes zoster; ID: infectious diseases; IQR: interquartile range; NSAIDs: non-steroidal anti-inflammatory drugs; OR: odds ratio; PCR: polymerase chain reaction; PHN: post-herpetic neuralgia; PMC: pain management clinic; PNS: peripheral nervous system; SD: standard deviation; VZV: varicella-zoster virus; ZAP: zoster-associated pain.

## Competing interests

The authors declare that they have no competing interests.

## Authors' contributions

GP, CC, CG, CDA and LP conceived and designed the study. GP, MT EP and LM performed most of the analyses on collected data, suggested and elaborated most of data interpretation criteria, and drafted the manuscript. CR, FS, AC, AA, FDM, GC and LP contributed to the study design, contributed substantially to the acquisition of data and to medical care of the enrolled patients. EP, LC, LC and DDA carried out most of laboratory assays. LM and GP performed most of the final statistical analyses; GP, LM and EP relevantly contributed in manuscript revision. AV was involved in critical evaluation, interpretation and discussion of data, as well as in critical revision of the final manuscript and its revision. The VZV Pain Study Group made substantial contributions in designing the study, enrolling patients, collecting data, and carrying out the educational phase of the protocol. All authors read and approved the final version of the present manuscript.

## Pre-publication history

The pre-publication history for this paper can be accessed here:

http://www.biomedcentral.com/1741-7015/8/58/prepub
